# An abdominal giant lipoma that was surgically resected: case report

**DOI:** 10.3389/fsurg.2025.1639098

**Published:** 2025-11-20

**Authors:** Jianchun Fan, Chunbaixue Yang, Xinran Cao, Xueliang Wu

**Affiliations:** 1 Institute of Cancer, The First Affiliated Hospital of Hebei North University, Zhangjiakou, China; 2 Graduate School, Hebei North University, Zhangjiakou, China; 3 Clinical Laboratory, The First Affiliated Hospital of Hebei North University, Zhangjiakou, China; 4 General Surgery, The First Affiliated Hospital of Hebei North University, Zhangjiakou, China

**Keywords:** lipoma, case report, benign tumor, abdominal mass, surgical excision

## Abstract

**Background:**

Giant retroperitoneal lipomas are rare benign tumors that frequently present with nonspecific symptoms, leading to delayed diagnosis. This case highlights the importance of multidisciplinary evaluation and surgical management in treating large retroperitoneal masses.

**Patient presentation:**

A 42-year-old woman presented with right lower abdominal distension and occasional nausea. Computed tomographic (CT) imaging revealed a massive retroperitoneal fat-containing lesion (22 cm × 9.8 cm × 31 cm) compressing adjacent organs. Surgical resection via a transrectus approach removed a 4.86-kg encapsulated lipoma. Postoperative recovery was uncomplicated, and pathology confirmed a benign lipoma.

**Conclusions:**

Complete surgical excision remains the treatment of choice for giant retroperitoneal lipomas. Early imaging [CT/magnetic resonance imaging (MRI)] and differentiation from malignancies are critical. This case, representing one of the heaviest reported retroperitoneal lipomas (4.86 kg), demonstrates that curative resection is feasible for massive symptomatic masses, and contributes technical and outcome data to the limited literature on giant abdominal lipomas. It underscores the importance of long-term follow-up to monitor recurrence.

## 1 Background

A lipoma is a benign mesenchymal tumor composed of mature adipose tissue (1). Typically presenting as a soft, mobile subcutaneous nodule, it is most common on the trunk, neck, and proximal extremities (2). These slow-growing lesions are usually asymptomatic and require no treatment. Small intra-abdominal lipomas are asymptomatic and incidentally detected during imaging for unrelated conditions. However, larger tumors may produce nonspecific symptoms such as abdominal distension, vague pain, or occasional nausea; in severe cases, compression of adjacent organs may cause urinary frequency or bowel obstruction (3, 4). Surgical excision is curative and generally indicated for diagnostic confirmation, cosmetic concerns, or symptomatic relief.

Intra-abdominal lipomas, unlike the more common subcutaneous lipomas, are benign adipocytic tumors arising within the abdominal cavity, including sites such as the retroperitoneum, mesentery, greater omentum, and perivisceral fat (5, 6). Lipomas constitute 4%–5% of all benign tumors (7). However, due to their rarity, the incidence of intra-abdominal lipomas has not been reported. Within intra-abdominal benign tumors, lipomas are already uncommon, and giant intra-abdominal lipomas (≥10 cm) are even rarer, with only scattered case reports worldwide. Clinically, giant retroperitoneal lipomas are most frequently misdiagnosed as other abdominal or retroperitoneal masses, including well-differentiated liposarcomas (the most common malignant mimic), ovarian teratomas (in female patients), uterine fibroids (due to pelvic location), and renal/adrenal myelolipomas (because of overlapping fat density on imaging) (8). Notably, no pathognomonic symptoms distinguish benign lipomas from liposarcomas. Liposarcomas may show faster progression (such as sudden abdominal enlargement within weeks) or severe pain (from necrosis or nerve invasion), but these features are not specific. Therefore, imaging and histopathology remain the diagnostic gold standard. This rarity limits clinical familiarity, contributing to misdiagnosis and delayed intervention.

This case report describes a literature review to provide diagnostic and management insights. Its significance lies in three aspects: (1) the resected lipoma weighed 4.86 kg, among the heaviest intra-abdominal lipomas reported, expanding data on giant lipoma cases; (2) this report outlines diagnostic differentiation (from ovarian teratoma, liposarcoma, and fibroids using CT/MRI) and the surgical technique (transrectus abdominis resection), offering practical guidance for clinicians; (3) it demonstrates that curative resection is feasible for giant symptomatic intra-abdominal lipomas while emphasizing the role of early imaging and multidisciplinary evaluation.

## 2 Case presentation

### 2.1 Patient status

A 42-year-old woman from Zhangjiakou City, Hebei Province, presented on July 2, 2024, with intermittent right lower abdominal distension and mild pain without an obvious trigger. Symptoms were relieved after rest and partially alleviated by self-administered traditional Chinese medicine (specific medication unknown). The patient reported occasional postprandial nausea, with rare vomiting, and denied acid regurgitation, heartburn, epigastric burning, hematemesis, melena, dizziness, or blurred vision. The abdominal pain occurred intermittently, 2–3 times daily, lasting approximately 30 min per episode, and was rated 3/10 on the Visual Analog Scale (VAS). The patient sought care at a local hospital on July 4, 2024. Ultrasound revealed a meso-echoic abdominal–pelvic mass, and MRI demonstrated a large retroperitoneal fat-containing lesion. The patient was referred to the Department of Vascular and Glandular Surgery, First Affiliated Hospital of Hebei North University, for further evaluation. Abdominal mass resection was performed on July 16, 2024. Postoperative recovery was stable and uneventful.

Family/Social History: No family history of lipomatosis or hereditary tumors. The patient did not smoke or drink alcohol. Genetic testing was not performed because of the lesion's benign features.

### 2.2 Clinical examination

On admission, the abdomen was symmetrical and flat, without varicose veins, peristaltic waves, or abnormal intestinal patterns. The abdominal wall was soft, with mild right lower quadrant tenderness, rebound tenderness, and muscle tension. A mobile, soft mass measuring approximately 15 cm × 20 cm was palpable, without pulsation, fluctuation, or marked tenderness. The liver and spleen were not palpable below the costal margin. Murphy's sign was negative, and no tenderness was elicited at McBurney's point or tympanum. No shifting dullness or abnormal bowel sounds were detected. No inguinal lymphadenopathy was noted. Digital rectal examination (lithotomy position) revealed no mass, bleeding, or other abnormalities.

### 2.3 Clinical data

**Figure 1:**
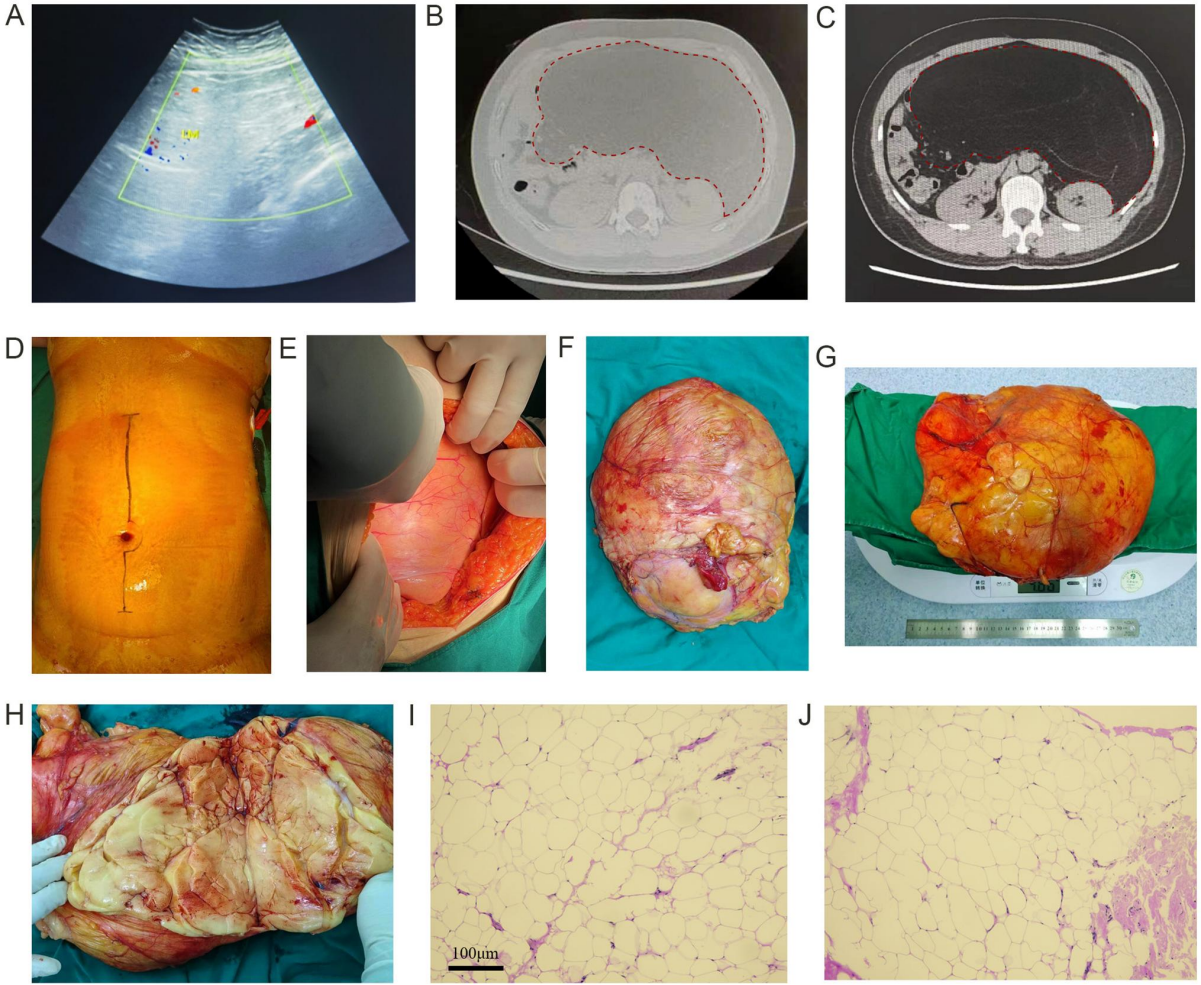
Imaging, intraoperative images, and gross and pathological features of giant lipomas. **(A)** Patient's B-mode ultrasound; **(B)**, **(C)** patient's abdominal noncontrast scan with enhanced scan; **(D)** Preoperative condition of the patient's abdomen, which was measured to be 104 cm; **(E)** condition of the lipoma seen at the time of intraoperative laparotomy; **(F)** gross full view of the lipoma (with its coating relatively intact); **(G)** weighing of the lipoma; **(H)** longitudinal anatomy of the lipoma.

Transabdominal uterine and adnexal ultrasound performed on July 6, 2024, at our hospital (Figure 1A) revealed: (1) Anteverted uterus, measuring 8.3 cm × 6.1 cm × 5.6 cm, with normal morphology, smooth contour, and uniform myometrial echogenicity; endometrium was 1.0 cm thick with homogeneous echotexture; intrauterine device (IUD) was appropriately positioned; cervix measured 3.1 cm. (2) Both ovaries were visualized. (3) A moderately echogenic pelvic mass measuring 23.0 cm × 28.0 cm × 15.0 cm, with ill-defined borders, homogeneous internal echogenicity, and punctate intralesional blood flow (Ovarian-Adnexal Reporting and Data System, category 3, O-RADS 3).

An abdominal non-contrast and contrast-enhanced scan (July 6, 2024, our hospital) revealed (Figures 1B,C): (1) A large fat-density mass shadow in the abdominopelvic cavity, likely retroperitoneal in origin, with internal septations. The lesion measured approximately 22 cm × 9.8 cm × 31 cm, compressed adjacent tissues, and demonstrated slight septal enhancement on contrast imaging. (2) A distended uterus with an IUD *in situ* and a small pelvic fluid collection.

Differential diagnosis: Ovarian teratoma (excluded due to absence of calcification/teeth on CT), liposarcoma (ruled out by homogeneous fat density and lack of nodular enhancement), and uterine fibroids (excluded given normal uterine visualization on MRI).

#### 2.3.1 Diagnostic challenges

The patient was initially misdiagnosed at a local hospital due to limited access to enhanced MRI for tissue characterization, reluctance toward surgical intervention, and nonspecific gastrointestinal-like symptoms.

Magnetic Resonance Imaging (MRI) at the local hospital also confirmed a massive retroperitoneal fat-containing lesion, consistent with the CT findings. However, the raw DICOM images were unavailable because, under the hospital's outpatient imaging policy, records are archived for only 6 months before deletion. Despite this limitation, the radiology report was accessible, and together with CT evidence of homogeneous fat density without nodular enhancement or soft tissue components, provided adequate information to differentiate the lesion from liposarcoma and other differentials in this case.

### 2.4 Surgical procedure

Under general anesthesia, the patient was positioned supine, routinely disinfected, and draped. A left abdominal trans-rectus abdominis incision, approximately 15 cm in length, was made to access the lesion. A left trans-rectus abdominis approach was selected to provide optimal exposure of the retroperitoneal space and facilitate *en bloc* resection of the large mass. The incision was made through the skin, subcutaneous tissue, anterior and posterior rectus sheaths, rectus abdominis muscle, and transversalis fascia, granting direct access to the retroperitoneum. This approach allowed sufficient working space for tumor dissection while minimizing injury to surrounding nerves and vessels. The skin, subcutaneous tissue, anterior and posterior sheath, rectus abdominis muscle, and rectus vaginalis were incised sequentially to enter the abdominal cavity for exploration. The mass was located behind the left peritoneum, extending superiorly to the lower margin of the pancreas, medially across the spine to the right renal hilum, and inferiorly to the uterorectal pouch. The lateral peritoneum was incised, and the mass was excised entirely using a combination of blunt and sharp dissection. No active bleeding was noted. A latex drainage tube was placed retroperitoneally through the left lower abdomen into the cavity. Intraoperatively, the mass was adherent to the posterior peritoneum and adjacent to the duodenum, pancreas, and right ureter, necessitating meticulous dissection to avoid injury. No major bleeding or organ damage occurred. Instrument and dressing counts were correct. The abdominal wall was sutured in layers, and the specimen was sent for pathological evaluation. The excised mass, encased in a relatively intact capsule, appeared yellowish with focal punctate hemorrhage on longitudinal sectioning and weighed 4.86 kg (Figures 1D,H).

### 2.5 Hematoxylin–eosin staining results

Grossly, the specimen measured 22.5 cm × 28.0 cm × 15.0 cm, and was yellowish in section. Typical adipose tissue was observed, and punctate hemorrhage was observed locally. Gross examination revealed a well-circumscribed mass with a smooth external surface and a thin, relatively intact fibrous capsule. The cut surface was homogeneous, yellow-tan, soft, and greasy with a lobulated architecture. No areas of necrosis or hemorrhage were present. Microscopically, the tumor consisted of mature adipocytes arranged in lobules separated by delicate fibrous septa. The adipocytes were uniform in size and shape, with small, peripherally located nuclei and no atypia. Lipoblasts, nuclear pleomorphism, and mitotic figures were absent. Specifically, there was no evidence of nuclear atypia, necrosis, or increased mitotic activity. The fibrous septa were thin and lacked atypical spindle cells. The capsule was intact, with no evidence of tumor infiltration. These features were consistent with a benign lipoma.

Given the classic histological features and absence of atypical elements, immunohistochemical staining was considered unnecessary and was not performed.

To exclude well-differentiated liposarcoma (WDLPS), extensive gross sampling was performed, focusing on any firm, nodular, or heterogeneous areas. Nine representative sections were obtained from different tumor regions, including the capsule and areas with atypical appearance, and all were submitted for histopathological examination. No solid, nodular, or necrotic foci were identified grossly or microscopically, supporting the diagnosis of benign lipoma.

Although immunohistochemical staining (such as Mouse Double Minute 2 homolog [MDM2] and Cyclin-Dependent Kinase 4 [CDK4) or molecular testing [such as MDM2 amplification by Fluorescence *in situ* Hybridization (FISH)] can aid in distinguishing WDLPS from lipoma, these were deemed unnecessary due to classic histological features: uniform adipocyte size, absence of lipoblasts, nuclear atypia, mitotic figures, and necrosis, along with the lesion's well-circumscribed nature and lack of radiological suspicion. Extensive sampling confirmed histological homogeneity without atypical areas.

The pathological diagnosis was consistent with a benign abdominal lipoma (Figures 1I,J).

### 2.6 Intervention tolerability

Adherence was confirmed by direct surgeon assessment during wound checks. The patient reported mild postoperative pain [Visual Analog Scale (VAS 2/10)], controlled with oral Nonsteroidal Anti-Inflammatory Drugs (NSAIDs), with no analgesic requirement beyond Day.

### 2.7 Follow-up

Telephone follow-up at 1 and 3 months postoperatively confirmed complete resolution of bloating and nausea. At 3 months, local abdominal ultrasound examination showed no recurrence or complications. The patient reported significant improvement in quality of life.

## 3 Patient perspective

During the 3-month follow-up, the patient shared her experience: “Before the surgery, the large abdominal mass caused persistent discomfort that significantly affected my daily life. I often felt a heavy sensation in the right lower abdomen, which made it difficult to perform routine activities such as walking for more than 10 min or bending over to pick up objects. Additionally, the occasional nausea and bloating disrupted my appetite and sleep quality, leading to increased anxiety about my health. Within 2 weeks after the tumor resection, the bloating and nausea completely subsided. By the fourth week, I was able to resume normal daily activities, including household chores and moderate exercise (e.g., walking for 30 min daily). I am particularly satisfied with the minimal surgical scar, which is well-hidden and does not cause cosmetic concerns. Throughout the treatment process, the medical team explained each step (from preoperative imaging evaluation to postoperative care) in a clear and patient manner, which greatly reduced my fear of surgery. Overall, this treatment has restored my physical function and quality of life, and I am very grateful for the professional care provided.”

## 4 Discussion

### 4.1 Comparison with previously reported cases

The novelty of our case was highlighted by comparing it with previously published cases of giant retroperitoneal lipomas using key clinical and pathological parameters.

Unlike the omental/mesenteric lipomas described by Saleem et al., Saoussen et al., and Azhar et al., or the lipoleiomyoma (mixed tumor) reported by Schaefer et al., our case originated in the retroperitoneum—a more concealed site, predisposed to deep organ compression. Its weight (4.86 kg) and size (22 cm × 9.8 cm × 31 cm) exceeded those reported by Saleem et al. (2.45 kg) and Saoussen et al. (8 cm), and represented a pure lipoma (unlike Schaefer et al.'s mixed lesion).

Our case utilized multimodal imaging (ultrasound + CT + MRI) with cross-validation (such as MRI fat-suppression sequences and the absence of nodular enhancement on CT) to exclude liposarcoma. This approach was superior to the single-modality imaging (CT only in Saleem et al.) or inconclusive biopsy (Azhar et al.) reported in prior cases.

Furthermore, our pathological evaluation—demonstrating the absence of lipoblasts, nuclear atypia, and capsular infiltration—more rigorously excluded lipoma-like liposarcoma than the descriptions in several referenced reports (Table 1). Finally, postoperative follow-up at 1 and 3 months completed the clinical course, providing a valuable paradigm for managing giant benign retroperitoneal lipomas.

**Table 1:** Comparison table of past lipoma cases.

Gender	Age	Tumor size/weight	Imaging modalities	Surgical management	Histopathological work-up	Outcomes/recurrence status	Reference
Male	61	23 × 18 × 7 cm, 2.45 kg	CT, USG-guided biopsy	Laparoscopic excision	Mature adipocytes, fat necrosis, no atypia	Uneventful, no recurrence	(5)
Male	43	14 cm (length)	CT, MRI	Open surgical resection	Mature adipocytes, no atypia or malignancy	No recurrence reported	(9)
Male	11 mo	30 × 19 × 12 cm	CT, USG, Trucut biopsy	Laparotomy with ileal resection	Mature adipocytes, no atypia	Well at 6 months, no recurrence	(10)
Female	45	30 × 20.5 × 12.5 cm	CT, USG-guided core biopsy	Exploratory laparotomy, *en bloc* resection	Adipocytes + smooth muscle, ER+, SMA+, Desmin+, HMB-45−	Resolved symptoms, no recurrence	(9)

Recent studies emphasize that WDLPS can closely mimic benign lipoma histologically. Although immunohistochemistry (such as MDM2 and CDK4) and FISH for MDM2 amplification are valuable for confirming WDLPS, they are not routinely warranted when both imaging and histology show unequivocal benign features (11). In our case, the absence of nuclear atypia, lipoblasts, septal thickening, or nodular enhancement on imaging strongly supported a diagnosis of lipoma. However, in resource-rich settings, molecular confirmation may provide additional assurance, particularly for large or deep-seated tumors.

### 4.2 Relapse and follow-up

The prognosis of lipoma is excellent, with recurrence rates under 5%, usually due to incomplete resection (12). Residual capsule or tumor fragments remain the primary risk factors. Accordingly, we recommend structured surveillance for giant retroperitoneal lipomas: abdominal ultrasound or CT at 1, 3, and 6 months postoperatively to detect early recurrence, followed by annual imaging (ultrasound or low-dose CT) for at least 5 years.

Conversely, abdominal liposarcoma carries a 30% mortality rate and a 50% recurrence rate. Reported survival ranges from 6 to 20 years, depending on prognostic factors (13). Recurrence is frequently earlier (within 1–2 years), multifocal, or invasive, requiring more frequent follow-up (CT every 3 months for the first 2 years, then every 6 months for 3–5 years) and potential reoperation or adjuvant therapy. In our patient, complete resection and benign histology were achieved. At the 3-month follow-up, no recurrence was observed, and annual imaging will continue as per the recommended schedule.

### 4.3 Conclusion

Lipomas originate from mature adipocytes and are pathologically characterized by aggregates of mature adipocytes encased in thin fibrous capsules. They may vary in size, occur throughout the body, and present at any age, from infancy to old age (14). Abdominal giant lipomas are rare but clinically significant solid tumors (15). While most lipomas arise in the waist, back, or limbs, those in the pelvis are frequently detected late because of their concealed location, lack of early symptoms, nonspecific clinical manifestations, and frequent confusion with other conditions (such as ovarian teratoma or uterine fibroids). Limited health check-ups further delay diagnosis, allowing the formation of large masses. Study limitations include short-term follow-up (3 months), absence of genetic testing for familial lipomatosis, and single-center experience without a comparative cohort.

The exact etiology of lipomas remains unclear, though genetic predisposition and lifestyle are implicated (6). Although lipomas can be detected via palpation, imaging—ultrasound, CT, or MRI—is frequently required for definitive diagnosis, sizing, and anatomical assessment (4). With few reports of giant abdominal lipomas, surgical excision remains the standard treatment (16). Early postoperative monitoring is critical to assess wound healing and detect complications, followed by long-term surveillance to evaluate recurrence and ensure durable outcomes.

Clinically, this case underscores the diagnostic overlap between giant retroperitoneal lipomas and liposarcomas. Symptoms such as abdominal distension or pain are nonspecific, while imaging findings such as fat density may mimic WDLPS (11). Misdiagnosis has profound implications: radical resection of a benign lipoma increases surgical morbidity, whereas incomplete resection of liposarcoma promotes recurrence. Reported recurrence rates for deep lipomas range from 3% to 62% (17). Therefore, accurate diagnosis and complete excision are essential. Furthermore, we believe that MDM2/CDK4 immunohistochemistry and FISH assay for MDM2 amplification are valuable tools for conclusively ruling out well-differentiated liposarcoma (WDLPS), particularly in diagnostically challenging cases (11). Their inclusion would undoubtedly strengthen diagnostic confidence. In the present case, however, the combined evidence from multimodal imaging (showing homogeneous fat density without nodular enhancement or thick septa) and extensive histopathological sampling (revealing uniform mature adipocytes without any atypia, lipoblasts, or suspicious solid areas) provided a compelling and consistent picture of a benign lipoma. Within our institutional context and given these unequivocal benign features, ancillary molecular testing was deemed not mandatory for diagnosis. Nevertheless, we recognize that in settings with sufficient resources, or for any large deep-seated lipomatous tumor, such confirmatory tests can provide an additional layer of diagnostic security and are recommended when there is any clinical or pathological uncertainty.

This case illustrates two significant clinical challenges. First, distinguishing lipomas from liposarcomas—both appear as large retroperitoneal fat-containing masses on CT/MRI, frequently with overlapping features such as homogeneous fat density (in WDLPS). In this case, the absence of nodular enhancement (on CT) and heterogeneous signal (on MRI) supported a benign diagnosis, but these findings are not definitive. Second, surgical implications of misdiagnosis: lipomas require only complete resection, while liposarcomas necessitate aggressive management with wider margins or adjuvant therapy. Misinterpreting a lipoma as a liposarcoma risks unnecessary extensive surgery (such as an *en bloc* resection involving adjacent organs), whereas the reverse risks recurrence. This case demonstrates that a multimodal approach—preoperative CT/MRI → intraoperative assessment of capsule integrity → postoperative pathology—is essential for accurate differentiation and optimal outcomes.

## Data Availability

The original contributions presented in the study are included in the article/Supplementary Material, further inquiries can be directed to the corresponding author.
