# Supplementary material for: An abdominal giant lipoma that was surgically resected: case report
Source: Front Surg. 2025 Nov 20;12:1639098. doi: 10.3389/fsurg.2025.1639098 (PMC12676285; doi:10.3389/fsurg.2025.1639098)
Supplement: Supplementary file 1 [file Datasheet1.pdf]

CARE Checklist of information to include when writing a case report

CARE case report guidelines

(cc) BY-NC-ND

Topic	Item	Checklist item description	Reported on Line
**Title**	1	The diagnosis or intervention of primary focus followed by the words "case report"	Title
**Key Words**	2	2 to 5 key words that identify diagnoses or interventions in this case report, including "case report"	Key Words
**Abstract (no references)**	3a	Introduction: What is unique about this case and what does it add to the scientific literature?	Abstract
	3b	Main symptoms and/or important clinical findings	Abstract
	3c	The main diagnoses, therapeutic interventions, and outcomes	Abstract
	3d	Conclusion—What is the main "take-away" lesson(s) from this case?	Abstract
**Introduction**	4	One or two paragraphs summarizing why this case is unique (**may include references**)	1. Background
**Patient Information**	5a	De-identified patient specific information.	2.1 Patient status
	5b	Primary concerns and symptoms of the patient.	2.1 Patient status
	5c	Medical, family, and psycho-social history including relevant genetic information	2.1 Patient status
	5d	Relevant past interventions with outcomes	2.1 Patient status
**Clinical Findings**	6	Describe significant physical examination (PE) and important clinical findings.	2.2 Clinical examination and 2.3 Clinical data
**Timeline**	7	Historical and current information from this episode of care organized as a timeline	2.1 Patient status
**Diagnostic Assessment**	8a	Diagnostic testing (such as PE, laboratory testing, imaging, surveys).	2.3 Clinical data
	8b	Diagnostic challenges (such as access to testing, financial, or cultural)	2.3 Clinical data
	8c	Diagnosis (including other diagnoses considered)	2.3 Clinical data
	8d	Prognosis (such as staging in oncology) where applicable	4.2 Relapse and Follow-up
**Therapeutic Intervention**	9a	Types of therapeutic intervention (such as pharmacologic, surgical, preventive, self-care)	2.4 Surgical procedure
	9b	Administration of therapeutic intervention (such as dosage, strength, duration)	2.4 Surgical procedure
	9c	Changes in therapeutic intervention (with rationale)	Not applicable
**Follow-up and Outcomes**	10a	Clinician and patient-assessed outcomes (if available)	2.7 Follow-up and 3. Patient Perspective
	10b	Important follow-up diagnostic and other test results	2.7 Follow-up
	10c	Intervention adherence and tolerability (How was this assessed?)	2.6 Intervention tolerability
	10d	Adverse and unanticipated events	None reported
**Discussion**	11a	A scientific discussion of the strengths AND limitations associated with this case report	4. Discussion
	11b	Discussion of the relevant medical literature **with references**.	4. Discussion
	11c	The scientific rationale for any conclusions (including assessment of possible causes)	4. Discussion
	11d	The primary "take-away" lessons of this case report (without references) in a one paragraph conclusion	4.3 Conclusion
**Patient Perspective**	12	The patient should share their perspective in one to two paragraphs on the treatment(s) they received	3. Patient Perspective
**Informed Consent**	13	Did the patient give informed consent? Please provide if requested	**Yes** ☑ **No** ☐
